# A few sequence polymorphisms among isolates of Maize bushy stunt phytoplasma associate with organ proliferation symptoms of infected maize plants

**DOI:** 10.1093/aob/mcw213

**Published:** 2016-12-10

**Authors:** Zigmunds Orlovskis, Maria Cristina Canale, Mindia Haryono, João Roberto Spotti Lopes, Chih-Horng Kuo, Saskia A. Hogenhout

**Affiliations:** 1 John Innes Centre, Department of Cell and Developmental Biology, Norwich Research Park, Norwich NR4 7UH, UK; 2 Luiz de Queiroz College of Agriculture, University of São Paulo, Department of Entomology and Acarology, Piracicaba 13·418-900, Brazil; 3 Institute of Plant and Microbial Biology, Academia Sinica, Taipei 11529, Taiwan

**Keywords:** Obligate biothroph, plant–microbe interactions, plant defence, effector biology, virulence genes, vector-borne plant pathogens, genome-wide association study

## Abstract

**Background and Aims** Maize bushy stunt phytoplasma (MBSP) is a bacterial pathogen of maize (*Zea mays* L.) across Latin America. MBSP belongs to the 16SrI-B sub-group within the genus ‘*Candidatus* Phytoplasma’. MBSP and its insect vector *Dalbulus maidis* (Hemiptera: Cicadellidae) are restricted to maize; both are thought to have coevolved with maize during its domestication from a teosinte-like ancestor. MBSP-infected maize plants show a diversity of symptoms. and it is likely that MBSP is under strong selection for increased virulence and insect transmission on maize hybrids that are widely grown in Brazil. In this study it was investigated whether the differences in genome sequences of MBSP isolates from two maize-growing regions in South-east Brazil explain variations in symptom severity of the MBSP isolates on various maize genotypes.

**Methods** MBSP isolates were collected from maize production fields in Guaíra and Piracicaba in South-east Brazil for infection assays. One representative isolate was chosen for *de novo* whole-genome assembly and for the alignment of sequence reads from the genomes of other phytoplasma isolates to detect polymorphisms. Statistical methods were applied to investigate the correlation between variations in disease symptoms of infected maize plants and MBSP sequence polymorphisms.

**Key Results** MBSP isolates contributed consistently to organ proliferation symptoms and maize genotype to leaf necrosis, reddening and yellowing of infected maize plants. The symptom differences are associated with polymorphisms in a phase-variable lipoprotein, which is a candidate effector, and an ATP-dependent lipoprotein ABC export protein, whereas no polymorphisms were observed in other candidate effector genes. Lipoproteins and ABC export proteins activate host defence responses, regulate pathogen attachment to host cells and activate effector secretion systems in other pathogens.

**Conclusions** Polymorphisms in two putative virulence genes among MBSP isolates from maize-growing regions in South-east Brazil are associated with variations in organ proliferation symptoms of MBSP-infected maize plants.

## INTRODUCTION

Phytoplasmas are obligate biotrophic plant pathogenic bacteria that replicate intracellularly in phloem sieve elements of infected plants. Phytoplasmas cannot be cultured *in vivo* in the laboratory, and depend on phloem-feeding hemipteran insects for transmission to plants (Weintraub and Beanland, 2006). Phytoplasmas infect a diversity of dicot and monocot wild, ornamental and crop plant species, and cause significant crop yield losses worldwide. For example, ‘flavescence doreé’ and ‘bois noir’ phytoplasmas devastate grapevine production in Europe (Botti and Bertaccini, 2007), and Maize bushy stunt phytoplasma (MBSP) has a high incidence and frequent outbreaks in maize fields across Latin America, resulting in high yield losses (Triplehorn and Nault, 1985; Bedendo *et al.*, 2000). Symptoms of phytoplasma-infected plants are indicative of modulation of fundamental plant developmental processes, including that of flower development and fruit production, contributing to losses in yield and seed production (Sugio *et al.*, 2011*b*). Phytoplasma whole-genome sequences are key to the identification of virulence genes responsible for developmental changes in host plants and the cause of yield losses.

Because phytoplasmas cannot be cultured, these bacteria are classified based on their 16S ribosomal (16Sr) DNA sequences into 16Sr group and sub-groups (Lee *et al.*, 2000). To date, there are only five fully assembled genomes (Oshima *et al.*, 2004; Bai *et al.*, 2006; Kube *et al.*, 2008; Tran-Nguyen *et al.*, 2008; Andersen *et al.*, 2013) and a further nine draft genome (contig) sequences available of phytoplasmas (Saccardo *et al.*, 2012; Chung *et al.*, 2013; Chen *et al.*, 2014; Kakizawa *et al.*, 2014; Mitrovic *et al.*, 2014; Quaglino *et al.*, 2015). These belong to 16Sr-I, -II, -III, -X and -XII groups of diverse clades within the phylogenetic tree (Hogenhout *et al.*, 2008), enabling assessment of the diversity in phytoplasma genome content and organization. Phytoplasma belong to the class Mollicutes, characterized by an absence of cell wall and small genomes (600–960 kb), as a result of genome reduction. Reduced genome size and absence of basal metabolic pathway genes reflect their dependency on their hosts for nutrients (Oshima *et al.*, 2004; Bai *et al.*, 2006). In addition, some phytoplasmas have lost DNA repair genes, such as *recA* (Bai *et al.*, 2006; Chu *et al.*, 2006). Despite their reduced metabolic capacity, phytoplasma genomes are rich in repeats, which can be > 20 kb (Bai *et al.*, 2006; Jomantiene and Davis, 2006; Wei *et al.*, 2008; Toruño *et al.*, 2010; Andersen *et al.*, 2013; Chung *et al.*, 2013; Ku *et al.*, 2013). The repeats are organized in conserved gene clusters named potential mobile units (PMUs) or sequence-variable mosaics (SVMs) (Bai *et al.*, 2006; Jomantiene and Davis, 2006). At least one PMU was shown to exist as chromosomal and extrachromosomal units in the genome of Aster Yellows phytoplasma Witches’ Broom (AY-WB) (Toruño *et al.*, 2010). PMUs are prone to recombination and degeneration, and there is evidence that the PMUs recombine and have horizontally transferred between diverged phytoplasmas (Bai *et al.*, 2006; Hogenhout and Seruga Musić, 2009; Sugio and Hogenhout, 2012; Chung *et al.*, 2013; Ku *et al.*, 2013). Interestingly, AY-WB PMU1 encodes several membrane-targeted proteins, which may function as conjugation systems for horizontal gene transfer between phytoplasmas or are virulence factors responsible for interactions of phytoplasma cells with plant or insect host factors (Toruño *et al.*, 2010). PMUs appear to determine the genome size reduction and plasticity of the small and AT-rich phytoplasma genomes (Bai *et al.*, 2006; Jomantiene and Davis, 2006; Hogenhout and Seruga Musić, 2009; Andersen *et al.*, 2013). Synteny of the circular chromosomes between closely related phytoplasmas within the ‘*Candidatus* Phytoplasma asteris’ (Ca. P. asteris) 16Sr-I group is low compared with that of other closely related bacteria (Hogenhout and Seruga Musić, 2009) and absent between phytoplasmas from different 16Sr groups. Also, some phytoplasmas, such as apple proliferation phytoplasma ‘*Ca*. P. mali’, have linear chromosomes (Kube *et al.*, 2008). Given the high number of repeats and differential loss of metabolic genes, including DNA repair genes, we hypothesized that phytoplasma isolates within a 16Sr (sub)-group are divergent in genome sequence.

The majority of phytoplasma virulence proteins (effectors) lie within or adjacent to PMU and PMU-like gene clusters (Bai *et al.*, 2009; Toruño *et al.*, 2010). Functional characterizations are available for effectors tengu-su, SAP11 and SAP54/phyllogen from AY-WB (16SrIA sub-group) and Onion Yellows phytoplasma strain M (OY-M; 16SrIB sub-group) (Sugio *et al.*, 2011*a*, *b*; Sugawara *et al.*, 2013; Lu *et al.*, 2014; MacLean *et al.*, 2014; Maejima *et al.*, 2014; Minato *et al.*, 2014; Orlovskis *et al.*, 2015; Orlovskis and Hogenhout, 2016). The SAP11 and SAP54 effectors induce stem proliferation and leaf-like flowers that resemble witch’s broom and phyllody symptoms, respectively, of phytoplasma-infected plants (Bai *et al.*, 2009; Hoshi *et al.*, 2009; MacLean *et al.*, 2011; Sugio *et al.*, 2011*a*). Phytoplasma effectors SAP11 and SAP54/phyllogen act by interacting with specific plant transcription factors and degrade/destabilize them, leading to the changes in leaf, stem and flower development, and the downregulation of defence responses that promote attraction of phytoplasma insect vectors (Sugio *et al.*, 2011*a*, *b*; Lu *et al.*, 2014; MacLean *et al.*, 2014; Maejima *et al.*, 2014; Orlovskis *et al.*, 2015; Orlovskis and Hogenhout, 2016).

Maize bushy stunt phytoplasma is predominantly transmitted by the maize specialist leafhopper, *Dalbulus maidis* (DeLong and Wolcott) (Hemiptera: Cicadellidae) (Nault, 1980; Oliveira *et al.*, 2011). Both the pathogen and the vector are present throughout maize production zones in Central and South America (Triplehorn and Nault, 1985; Oliveira *et al.*, 2013; Van Nieuwenhove *et al.*, 2015) and are thought to have coevolved with maize since its domestication from a teosinte ancestor (Nault, 1980; Nault and DeLong, 1980; Doebley *et al.*, 1997). Maize bushy stunt disease symptoms are characterized by leaf reddening, shortening of internodes, plant height reduction (stunting), lower grain yield and lateral shoot production (Nault, 1980). MBSP-infected maize plants show a diversity of symptoms, depending on maize genotype, weather conditions and perhaps also the MBSP isolate (Murral *et al.*, 1996; Moya-Raygoza and Nault, 1998), and it is likely that MBSP is under strong selection for increased virulence and insect transmission on the maize genotypes that are widely grown in Brazil. Observed symptom variations include differences in the severity of organ proliferations. Therefore, we hypothesized that variation in the presence and sequence of SAP11 homologues among MBSP isolates may explain differences in organ proliferation symptoms.

In this study, we collected MBSP isolates from two maize-growing regions in Brazil and found isolates that differ in the induction of lateral branching in infected maize. To sequence whole genomes of the MBSP isolates, we made use of the recent discovery that whole phytoplasma genomes can be recovered and assembled by sequencing phytoplasma-carrier insects. We assembled the genome of MBSP isolate M3 (16Sr-IB) into one circular chromosome and compared it with the complete genomes of AY-WB (16Sr-IA) and OY-M (16Sr-IB) phytoplasmas of ‘*Ca*. P. asteris’. All three phytoplasmas have SAP11 homologues and several other candidate effectors, whereas no SAP54/phyllogen homologue was identified in MBSP. The candidate effector genes often lie within or near PMUs in the MBSP genome. Nine out of 86 polymorphisms among the Brazilian MBSP isolates associated with the lateral branching symptoms. One of these caused a frameshift mutation in a gene encoding a phase-variable lipoprotein, which is a candidate effector, and two other polymorphisms locate in a lipoprotein ABC exporter, whereas no polymorphisms were detected in other candidate effector proteins, including SAP11. A role for the lipoprotein and exporter in MBSP virulence is discussed.

## MATERIALS AND METHODS

### Sampling and maintenance of MBSP isolates

**Fig. 1.**
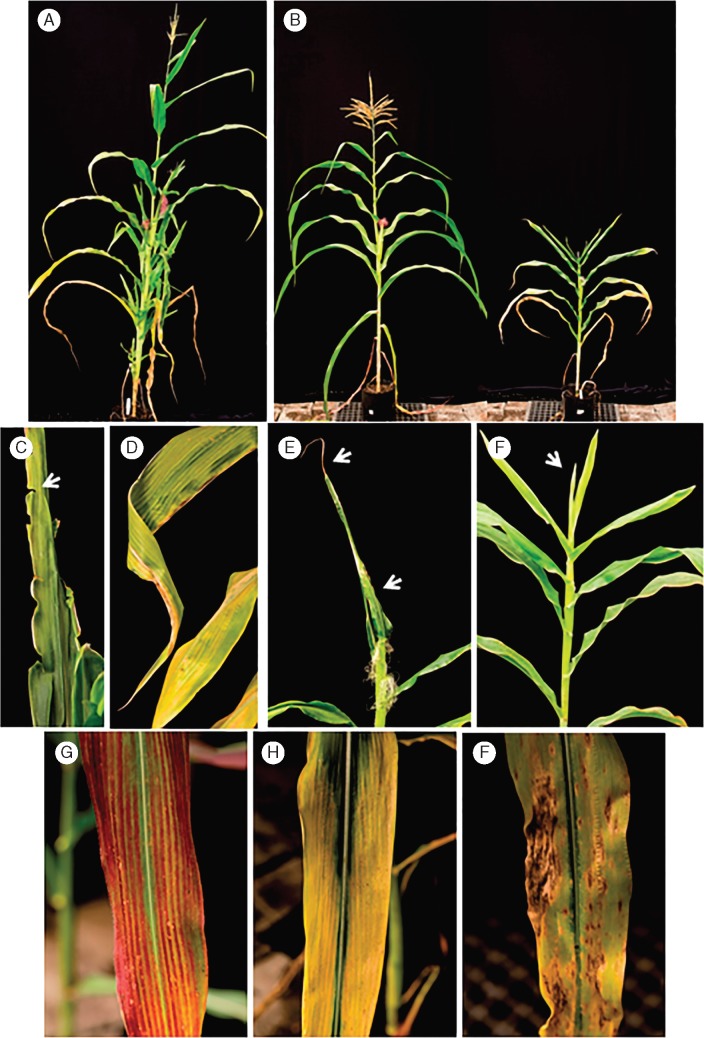
Maize bushy stunt phytoplasma (MBSP) induced diverse morphological changes in maize plants. (A) Infected plants demonstrate lateral branching and ear proliferation. (B) Plant stunting is a common symptom in infected maize (MBSP-infected plant on the right, non-inoculated control on the left). (C) Leaf rip (marginal tearing indicated by the white arrow). (D) Infected leaves curl (twist) and develop necrotic tissue at the tip. (E) Young leaves remain closed in the whorl and do not fully expand. (F) Young leaves (arrow) at the plant tip show characteristic chlorosis which is usually absent in older basipetal leaves. (G) Leaf reddening. (H) Leaf yellowing. (I) Necrotic lesions on the leaf blade. Pictures were taken 3 weeks after the inoculation with MBSP.

Whole maize plants exhibiting typical MBSP symptoms were sampled from maize fields in Brazil (Table 1, Fig. 1) and brought to the laboratory. About 200–300 MBSP-free third instar nymphs of *D. maidis* were confined on each plant and allowed to feed for a 2 d acquisition access period (AAP), followed by a 25 d latency period on healthy maize seedlings and then transferred to healthy test plants for a 3 d inoculation access period (IAP). MBSP isolates were maintained via cyclic leafhopper transmission to healthy maize plants as described above. Plants inoculated with different phytoplasma isolates were kept in separate screened cages in a greenhouse to prevent cross-inoculation.

**Table 1:** Collection date and sites of Maize bushy stunt phytoplasma isolates in Brazil

Reference	Locality (City, State)	Date of sampling	Altitude (m)	Latitude (S)	Longitude (W)
R4	Piracicaba, São Paulo	15 August 2012	565	22°70′	47°63′
G2	Guaíra, São Paulo	22 May 2013	512	20°32′	48°31′
M3	Piracicaba, São Paulo	29 May 2013	565	22°70′	47°63′
Bouquet	Guaíra, São Paulo	12 June 2013	512	20°32′	48°31′
T14	Piracicaba, São Paulo	21 June 2013	565	22°70′	47°63′
E10	Piracicaba, São Paulo	8 November 2013	565	22°70′	47°63′
G5	Sete Lagoas, Minas Gerais	22 May 2013	761	19°46′	44°24′

### Phenotypic analysis of maize symptoms

Different maize lines and hybrids were inoculated with a geographically diverse selection of MBSP isolates and analysed for changes in maize yield and disease symptom development. Two to six plants of maize lines named CRE1, CRE2 and CRE3 (Luiz de Queiroz College of Agriculture Maize Germplasm Bank), and the hybrids 30F35H (Pioneer™) and 2B433PW (Dow Agrosciences™) were vector-inoculated with four MBSP isolates (Supplementary Data Table S1). Plants were grown in 0·3 L pots containing soil mix (Topstrato Hortaliças HT, Vida Verde, Mogi Mirim, SP, Brazil) and inoculated 10 d after sowing. To obtain inoculative *D. maidis*, 450 insects were confined on phytoplasma source plants for a 3 d AAP and maintained on healthy plants for a 20 d latency period. The insects were then confined on healthy maize seedlings of the different lines/hybrids at the two-leaf stage for a 3 d IAP, using ten insects per seedling. Healthy plants never exposed to insects previously were used as negative controls. Inoculated and healthy plants were immediately transplanted to 5 L pots with the soil mix and kept in a greenhouse, where the plants received natural light. The experiment was carried out once during late summer and autumn (February–May 2014). We assessed the development of MBSP symptoms and measured quantitative traits related to the disease in maize, such as plant height, number of internodes and lateral branching. In addition, we scored symptoms such as reddening, yellowing and necrotic lesions in the leaves, and evaluated traits related to yield, such as number of cobs and cob weight.

### Statistical analysis of MBSP-infected maize phenotypes

Quantitative traits related to phytoplasma symptoms and yield of infected plants were analysed using analysis of variance (ANOVA) with Dunnet’s test for multiple pairwise comparisons with non-infected maize plants. General Linear Model (GLM) statistics were used to test effects of maize genotypes and MBSP isolates, as well as interactions between the two on variations in the severity of MBSP disease symptoms. Multivariate analysis on variance in the disease parameters [principal component analysis (PCA)] was performed with data of relative changes in height, lateral branching, cob weight and other morphological features in infected plants compared with non-infected controls to assess similarities among MBSP isolates and symptom development among various maize genetic backgrounds. Statistical analysis was performed using Minitab 16 Statistical Software^®^ (Minitab Inc., PA, USA).

### Isolation of MBSP DNA

For SAP11 genomic island assessment, DNA was extracted from field-collected MBSP-symptomatic maize plants using a DNeasy Plant Kit (Qiagen, Hilden, Germany) and from *D. maidis* fed from MBSP-infected maize using a DNeasy Blood and Tissue Kit (Qiagen), following the manufacturer’s instructions. DNA was extracted from established greenhouse isolates or insects that acquired the MBSP in the greenhouse, except G5, that was obtained directly from a field-infected plant (Table 1). Samples were tested for the presence of MBSP as described below.

Whole-genome sequences of MBSP isolates R4, M3, Bouquet, G2, E10 and T14 were obtained by sequencing MBSP-carrier *D. maidis*. For this, the leafhoppers were confined on MBSP source plants to acquire MBSP for 48 h, followed by a 3 week latency period on healthy plants. Individual insects were macerated with pestles individually in 1·5 mL microtubes, and extraction was conducted with the DNeasy Blood and Tissue Kit (Qiagen), according to the manufacturer’s instruction. DNA was eluted in 50 μL of distilled deionized water. A PCR to test the presence of MBSP in the insect DNA samples was conducted with the 16SrI-phytoplasma-specific P1/AYint primer pair to amplify a 1·5 kb fragment of the 16S rDNA gene (Supplementary Data Table S2). The PCR was performed with 1× PCR buffer (Invitrogen, Carlsbad, CA, USA), 1·5 mm MgCl_2_, 0·2 mm dNTP (Invitrogen), 0·5 mm of each primer and 1 U μL^–1^ of *Taq* DNA polymerase (Invitrogen), in a final reaction volume of 25 μL. The PCRs were carried in a Veriti 96 Well Thermal Cycler (Applied Technologies), settings: 2 min at 94 °C; 30 cycles of 1 min at 94 °C, 1 min at 56 °C and 2 min at 72 °C; and a finally 5 min at 72 °C. The reaction mixtures were separated by size by electrophoresis on a 1 % agarose gel, and the PCR products were visualized by UV transillumination. MBPS-positive DNA samples were pooled together and further cleaned up via a phenol:chloroform:isoamylalcohol (25:24:1) (Sigma, St Louis, MO, USA) treatment following standard procedures (Green and Sambrook, 2012).

### Sequencing SAP11 effector genes and genotyping SAP11 islands from MBSP isolates

Plant and pooled insect samples with 1·5 kb 16S rDNA amplification products were taken to conduct additional PCRs to assess the presence of SAP11 and adjacent genes of the SAP11 island that were observed in the partial genome sequence from a Mexican strain of MBSP (Sugio and Hogenhout, 2012) (Table S2). The PCRs were carried out with the following settings: 2 min at 94 °C; 40 cycles of 1 min at 94 °C, 30 s primer annealing temperatures and extension time depending on amplicon length at 72 °C (Table S2), followed by a final 5 min at 72 °C. All PCRs were separated by electrophoresis on a 1 % TBA (Tris-borate-EDTA) buffer agarose gel. The PCR products were purified using a QIAquick PCR purification kit (Qiagen) and both DNA strands were sequenced (Eurofins Genomics, Germany) using the ame amplification primers. The sequences were aligned using the Clustal Omega Multiple Sequence Alignment tool.

### Genome sequencing of MBSP isolates

The procedures for genome sequencing, assembly and annotation were based on those described elsewhere, including construction of a one paired-end library with a target insert size of approx. 550 bp and shotgun sequencing of each library on the Illumina MiSeq platform (Illumina) (Chung *et al.*, 2013; Chang *et al.*, 2015).

The strain M3 was selected as the representative for *de novo* genome assembly using Velvet v1.2.10 (Zerbino and Birney, 2008). The contigs were assigned as being of phytoplasma or plant origin based on BLASTX (Camacho *et al.*, 2009) searches against the NCBI non-redundant database (Benson *et al.*, 2015). The complete genome of AY-WB phytoplasma (Bai *et al.*, 2006) was used as a reference for scaffolding. Subsequently, PCR and Sanger sequencing were used for gap filling until the complete sequence of the entire circular chromosome was determined. For final verification, the Illumina reads were mapped to the assembly using Burrows–Wheeler Aligner (BWA) v0.7.12 (Li and Durbin, 2009), programmatically checked using the MPILEUP program in SAMTOOLS package v1.2 (Li *et al.*, 2009), and visually inspected using Integrative Genomics Viewer (IGV) v2.3.67 (Robinson *et al.*, 2011).

The programs RNAmmer (Lagesen *et al.*, 2007), tRNAscan-SE (Lowe and Eddy, 1997) and Prodigal (Hyatt *et al.*, 2010) were used for gene prediction. The gene names and product descriptions were first annotated based on the homologous genes in other phytoplasma genomes (Bai *et al.*, 2006; Chung *et al.*, 2013) as identified by OrthoMCL (Li *et al.*, 2003). Subsequent manual curation was based on BLASTP (Camacho *et al.*, 2009) searches against the NCBI non-redundant database (Benson *et al.*, 2015) and the KEGG database (Kanehisa and Goto, 2000; Kanehisa *et al.*, 2010).

Sequence polymorphisms among genomes of MBSP isolates were identified by mapping the Illumina reads from other MBSP strains to the complete chromosome sequence of MBSP strain M3 using BWA, MPILEUP and IGV as described above. All potential polymorphisms were manually inspected to remove false-positive calls. Subsequently, the confirmed polymorphic sites were examined in Artemis (Carver *et al.*, 2012) to identify the genetic features affected. The genome map was plotted using CIRCOS (Krzywinski *et al.* 2009). The pairwise genome alignments were generated using MUMmer v3.23 (Kurtz *et al.* 2004), the minimum match length (option ‘–l’) was set to 70 to reduce spurious hits.

### Nucleotide sequence accession numbers

Sequences of MBSP M3 have been deposited in the GenBank database under accession numbers SRR3348967 for Sequence Read Archive (SRA) and CP015149 for the complete genome. Other SRA accessions are SRR3354351 (MBSP R4), SRR3354352 (MBSP E10), SRR3354354 (MBSP G2), SRR3354355 (MBSP T14) and SRR3354356 (MBSP Bouquet).

### Single-marker regression analysis

PLINK v1.07 software (http://pngu.mgh.harvard.edu/purcell/plink/) developed by Purcell *et al.* (2007) was used to identify sets of polymorphic loci that share an identical allelic distribution among the four MBSP isolates for which symptoms were analysed and that have full genome sequence data, and to perform single-marker regression analysis to associate the allelic distribution among MBSP isolates in the sets of similar polymorphic loci with the quantitative traits of MBSP-induced disease symptoms.

## RESULTS

### Disease symptoms vary depending on MBSP isolate and maize genotype

**Fig. 2.**
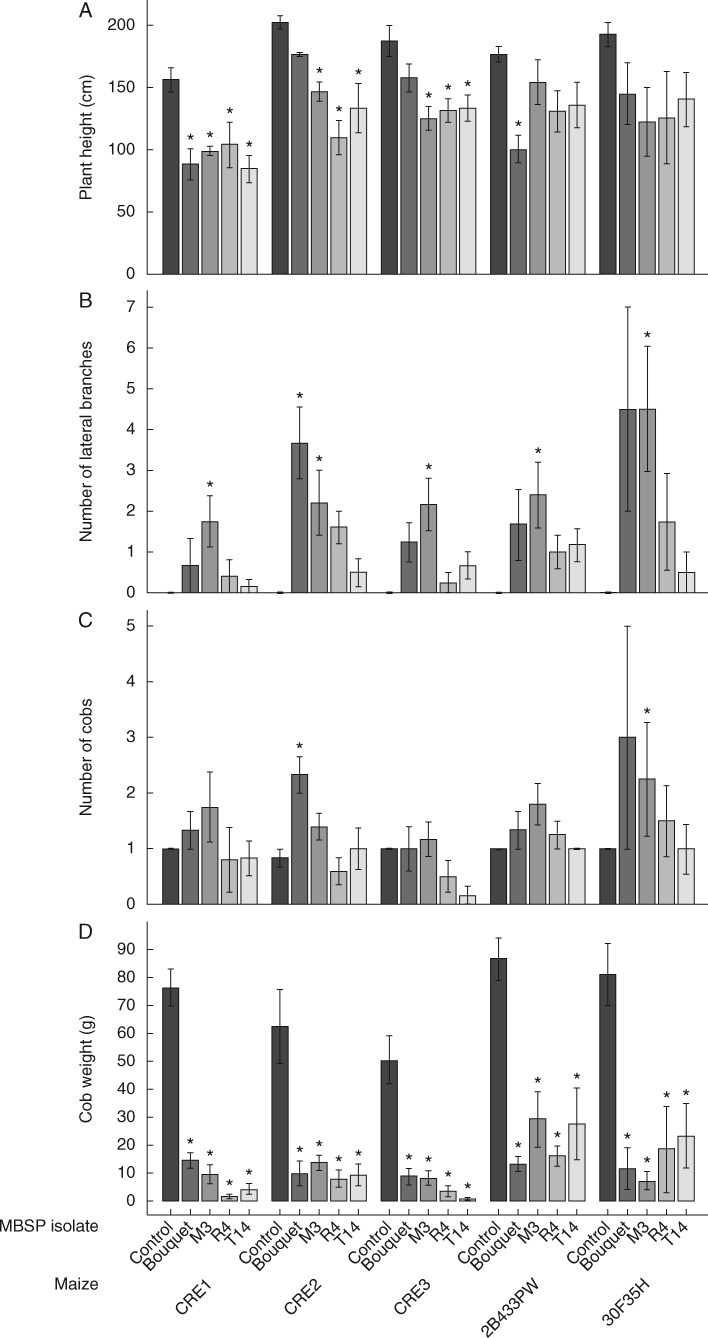
Maize bushy stunt phytoplasma (MBSP) reduces maize yield and induces proliferation of vegetative and female reproductive organs. Quantitative differences in plant height (A), lateral branching (B), number of cobs (C) and cob weight (D) are compared between infected and non-infected plants for five different maize genotypes in the greenhouse experiment. Significant differences from the control (non-infected) treatment within each maize background are indicated by an asterisk (ANOVA with Dunnet’s test for multiple pairwise comparisons with a reference). Column and bar indicate the mean and s.e.m., respectively.

**Fig. 3.**
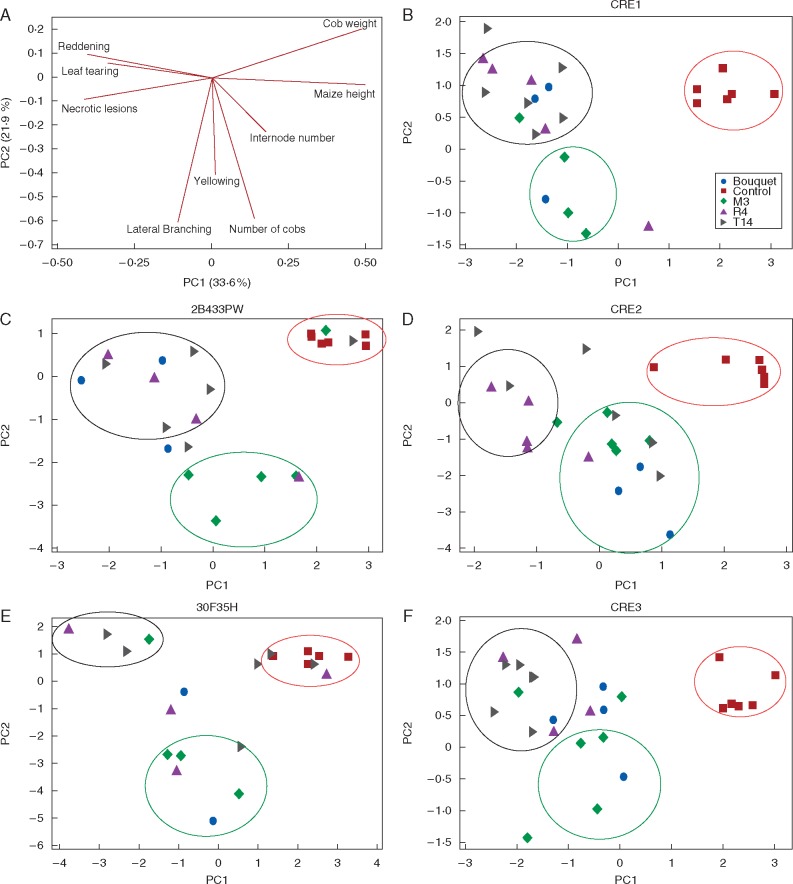
Principal components analysis (PCA) identifies correlated MBSP disease symptoms in maize and demonstrates variation of host responses to MBSP isolates in diverse maize genetic backgrounds. The eigenvector values of MBSP symptoms are plotted as part of principal components 1 (PC1) and 2 (PC2) and unravel co-ordinated groups of morphological changes induced by MBSP (A). MBSP isolates are grouped depending on their contribution to the disease symptoms included in PC1 (explaining 33·6 % of the variation) and PC2 (21·95 % of the variation) for three maize varieties (B, D, F) and two commercial hybrids (C, E). All non-infected control treatments show no reduction in height or cob weight and absence of disease symptoms.

To investigate the effect of MBSP isolate infection on disease development in maize, MBSP isolate Bouquet collected from a maize field in Guaíra and MBSP isolates R4, M3 and T14 from maize fields in Piracicaba (Table 1) were introduced into five maize genotypes that are susceptible to MBSP, i.e. CRE1, CRE2 and CRE3, 30F35H and 2B433PW (Table S1) via *D. maidis* transmission. Commercial maize hybrids 28433PW and 30F35H are widely grown in Brazil, including in maize fields in Paracicaba and Guaíra, where the MBSP isolates were collected. MBSP infections induced a diverse range of morphological changes in the different maize genotypes, including increased lateral shoots from the main stem (hereafter referred to as lateral branching) (Fig. 1A), stunting (decrease in stem height) (Fig. 1B), leaf tearing (Fig. 1C), leaf coiling (Fig. 1D), reduced leaf expansion (Fig. 1E), yellowing of young emerging leaves and fully emerged leaves (Fig. 1F, H), leaf reddening (Fig. 1G) and leaf necrosis (Fig. 1I). We quantified the effect of MBSP infection on plant height (cm), lateral branching, number of internodes, number of cobs, cob weight, as well as leaf reddening and yellowing (scale of notes rating from 0 for absence to 3 for severe) and presence/absence of necrotic lesions on the leaves (Fig. 2; Supplementary Data Fig. S1). MBSP isolate M3 had the most consistent increase in the number of lateral branches in the five maize genotypes among the MBSP isolates (Fig. 1B), whereas the other symptoms were more variable depending on MBSP isolate and maize genotype (Fig. 2A, C, D;Fig. S1). Analysis of the quantitative symptom data with GLM statistics showed that the MBSP isolate contributed significantly to variation in internode number, lateral shoot formation and cob number, and maize genotype contributed significantly to variation in lateral shoot formation, leaf reddening, leaf yellowing, leaf necrotic lesions and cob number, whereas the reductions in plant height and cob weight occurred independently of MBSP isolate and maize genotype (Table 2). PCA revealed that plant height is inter-correlated with cob weight, leaf reddening with leaf tearing and necrotic lesions, and lateral shoot formation with cob number production and leaf yellowing (Fig. 3A). The MBSP isolates contributed differentially to these symptom groups in each of the five maize genotypes, with MBSP isolate M3 having the most consistent correlation with the lateral branching, increase in cobs and leaf yellowing symptom group, and MBSP isolates R4 and T14 contributing most strongly to the leaf reddening, leaf tearing and necrosis symptom group (Fig. 3B–F). In conclusion, MBSP isolates contribute to symptom variations in all maize genotypes. In particular, MBSP isolate M3 infection shows the strongest effects on increased lateral branching among the maize genotypes.

**Table 2:** Summary statistics of General Linear Model (GLM) testing the effect of maize genotype and Maize bushy stunt phytoplasma (MBSP) isolate, as well as the interaction between the two factors on the variation in disease symptom severity in a fully crossed experimental design

Trait	Maize genotype		MBSP isolate		Genotype × MBSP isolate	
	*F*_4,71_	*P*-value	*F*_3,71_	*P*-value	*F*_12,71_	*P*-value
Plant height	1·24	0·302	0·33	0·804	1·03	0·428
Number of internodes	1·84	0·131	3·64	**0·017**	1·15	0·334
Lateral branching	1·91	**0·001**	11·14	**<0·001**	1·23	0·279
Leaf reddening	13·02	**<0·001**	2·45	0·07	2·20	**0·020**
Leaf yellowing	11·27	**<0·001**	0·68	0·565	1·36	0·206
Necrotic lesions	4·92	**0·001**	1·15	0·334	1·86	0·054
Number of cobs	3·70	**0·009**	6·35	**0·001**	0·60	0·853
Cob weight	1·96	0·110	0·43	0·733	0·82	0·632

Degrees of freedom for treatments and statistical error are indicated for each factor.

All treatments were treated as fixed factors.

Significant effects are indicated in bold.

### The sequence of the SAP11 effector is conserved among diverged MBSP isolates

Phytoplasmas produce specific effectors that interfere with plant development, including induction of lateral branches (witches’ broom) (Sugio *et al.*, 2011*a*) and leaf-like flowers (phyllody) (MacLean *at al.*, 2011, 2014). The SAP11 effector of AY-WB phytoplasma contributes to increased stem production in *Arabidopsis thaliana* and was shown to destabilize plant TCP (TEOSINTE BRANCHED1, CYCLOIDEA and PCF) transcription factors (Sugio *et al.*, 2011*a*) that are conserved among plant species (Cubas *et al.*, 1999) and includes the TCP transcription factor Teosinte branched (TB1), which regulates stem lateral branching and tillering (basal branches) in maize (Doebley *et al.*, 1997; Whipple *et al.*, 2011). We previously identified a SAP11 homologue in a Mexican strain of MBSP (Sugio and Hogenhout, 2012). Hence, we hypothesized that the differential contributions of MBSP isolates to branching induction in infected maize genotypes may be due to variation in SAP11 sequences among the MBSP isolates. To test this, we amplified the SAP11 gene and other genes previously identified to be located in the SAP11 PMU-like region in the Mexican strain (Supplementay Data Fig. S2B) from R4, M3, Bouquet and T14 and three additional isolates (E10, G2 and E10) (Table 1). All genes were successfully amplified and the SAP11 sequence was identical among the MBSP isolates from Brazil and also to one MBSP isolate from Mexico (Supplementary Data Fig. S2A). Thus, in contrast to our previous predictions, the variation in lateral branching symptoms among the MBSP isolates cannot be explained by sequence variations in the SAP11 sequences and the absence/presence of genes in the SAP11 PMU-like region.

### The whole-genome sequence of the MBSP isolate M3

**Fig. 4.**
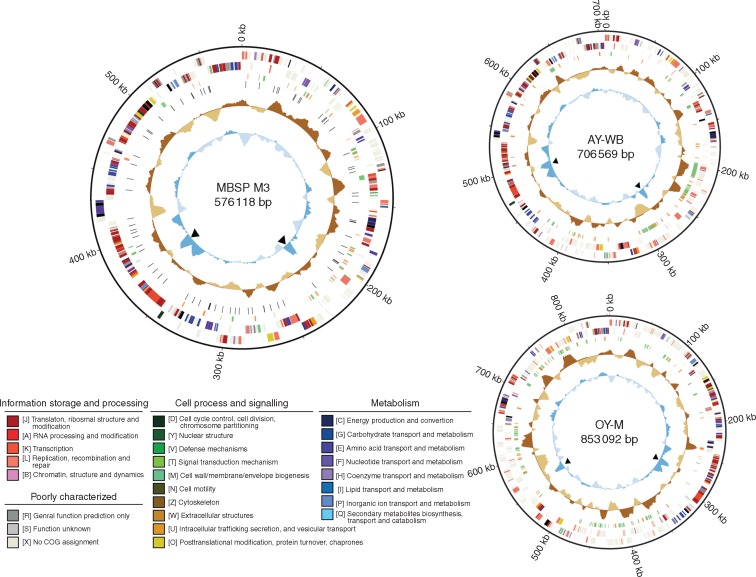
Genome maps of the three phytoplasmas belonging to the aster yellows group (16SrI). Concentric circles from the outside in: (1) scale marks, (2 and 3), protein-coding genes on the forward and reverse strand, respectively (color-coded by the functional categories), (4) putative effector (orange) and PMU (green) genes, (5) polymorphic sites among the MBSP strains (not applicable in AY-WB and OY-M), (6) GC skew (positive: dark brown; negative: light brown), and (7) GC content (above average: dark blue; below average: light blue); peaks corresponding to the rRNA gene clusters are highlighted by black triangles.

We sequenced the whole genome of MBSP isolate M3, because this isolate showed the most consistent induction of lateral branching. Genomic DNA was obtained from MBSP-carrier insects (see the Materials and Methods) and used for 2 × 300 MiSeq paired-end library preparation (±500 bp insert size). Of the total 2·4 million reads, 1·6 % (38 000 reads) were derived from the MBSP genome and assembled into five contigs. Gaps were closed by PCR and Sanger sequencing of the PCR products. Closure of all gaps and subsequent annotation revealed that the MBSP isolate M3 genome is 576 118 bp and encodes 531 genes (Fig. 4). The gene for chromosomal replication initiation protein DnaA was annotated as the first gene starting at base pair 1 and the gene for asparaginyl-tRNA synthetase AsnC as the last gene ending at base pair 575 086. A potential origin of replication (*oriC*) of the chromosome is probably located in the 1032 bp region between the *dnaA* and *asnC* genes, consistent with the protein-coding genes predominantly being located on the forward strand from 1 to 300 bp and on the reverse strand from approx. 300 to 575 kb, though stretches of protein-coding genes were also often located on the reverse strand between 125 and 300 kb. Nonetheless, the chromosome replication fork is likely to start upstream of *dnaA* and to terminate at approx. 300 kb.

**Fig. 5.**
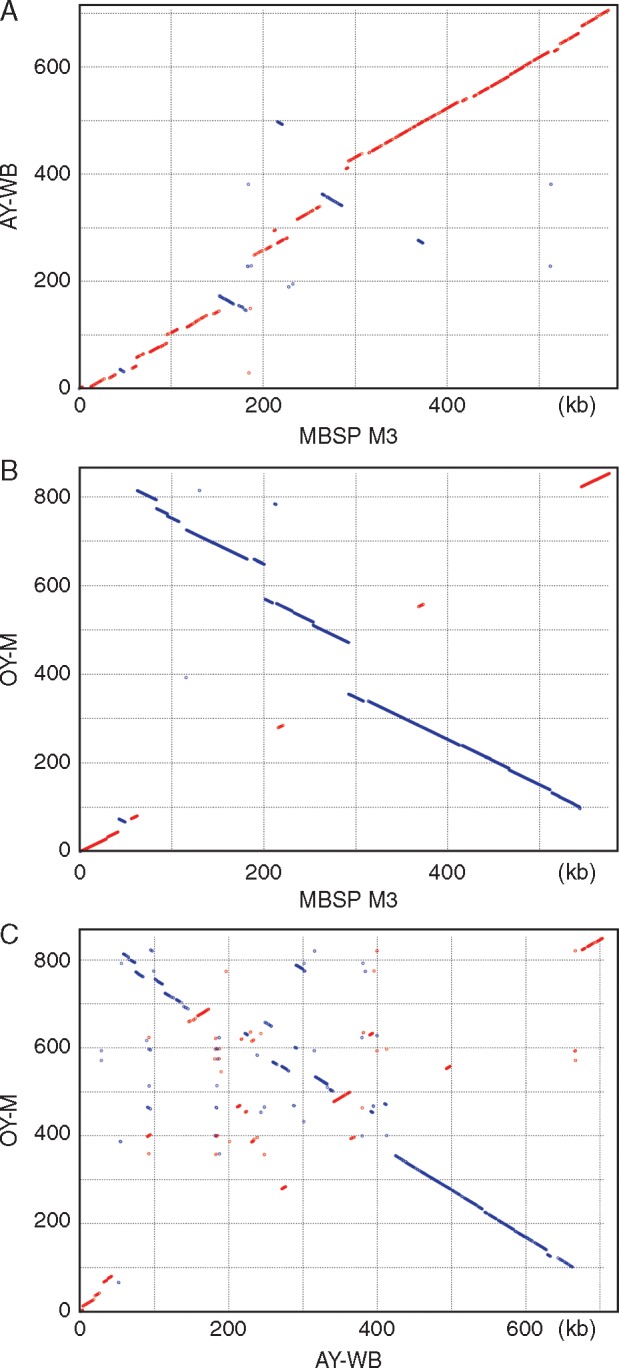
Pairwise genome alignments. Matches on the same strand are indicated by red dots, and matches on the opposite strands are indicated by blue dots.

The MBSP genome has an irregular GC-skew pattern (Fig. 4) that is different from most prokaryotic genomes, which usually consist of two major shifts near the origin of replication and the terminus of replication (Guy and Roten, 2004). However, the AY-WB and OY-M genomes also have irregular GC-skew patterns (Fig. 4) (Oshima *et al.*, 2004; Bai *et al.*, 2006) which is indicative of high genomic plasticity, possibly caused by relatively recent recombination events of, for example, PMUs (Bai *et al.*, 2006). Alignments of the MBSP, AY-WB and OY-M chromosomes showed better syntenies of the MBSP vs. AY-WB and MBSP vs. OY-M chromosomes compared with that of the AY-WB vs.OY-M chromosomes (Fig. 5). The majority of the metabolic genes lie in the 400–600 kb regions and the majority of the PMU-like sequences in the 150–400 kb regions of the AY-WB and MBSP phytoplasma chromosomes, whereas PMU-like sequences are distributed more throughout the genome in OY-M phytoplasma (Fig. 4). The PMU-rich regions show large inversions and discontinuous synteny that suggest recombinations have occurred in these regions since MBSP, AY-WB and OY-M diverged from their common ancestor.

The MBSP M3 isolate has 36 candidate effector genes (based on the presence of a signal peptide sequence and absence of a predicted transmembrane domain beyond the signal peptide sequence; Bai *et al.*, 2009) (Supplementary Data Table S3). Interestingly, PMU-like genes, which were identified based on similarities to *tra5*, *dnaB*, *dnaG* and other genes that are present in AY-WB PMU1 (Bai *et al.*, 2006), and effector genes co-localize between 0 and 250 kb on the genome map, but not in the 250–550 kb stretch of the MBSP genome (Fig. 4). Similarly, in the AY-WB genome, PMU-like and effector genes co-localize between 150 and 400 kb and less so in the other parts of the genome, whereas both groups of genes are distributed throughout the OY-M genome (Fig. 4).

Ten of the 36 MBSP effectors have homologues in other phytoplasmas; these include tengu-su and SAP11 (Table S3). However, no SAP54 homologue was identified in the MBSP genome. The SAP11 sequence was identical to that of the sequenced PCR product obtained earlier (Fig. S2A, B). Sixteen out of 36 MBSP candidate effector protein genes are located within or near to five predicted PMU-like regions (Supplementary Data Table S4) and six putative effector genes encoding homologues lie within or adjacent to MBSP_PMU3 (Fig. 7A, Table S4). SAP11 is also part of a PMU-like region in the MBSP isolate M3 genome (Table S4 and Fig. S2) and in a Mexican isolate of MBSP (Sugio and Hogenhout, 2012).

### Resequencing of the genomes of multiple MBSP isolates identified 86 polymorphic sites

**Fig. 6.**
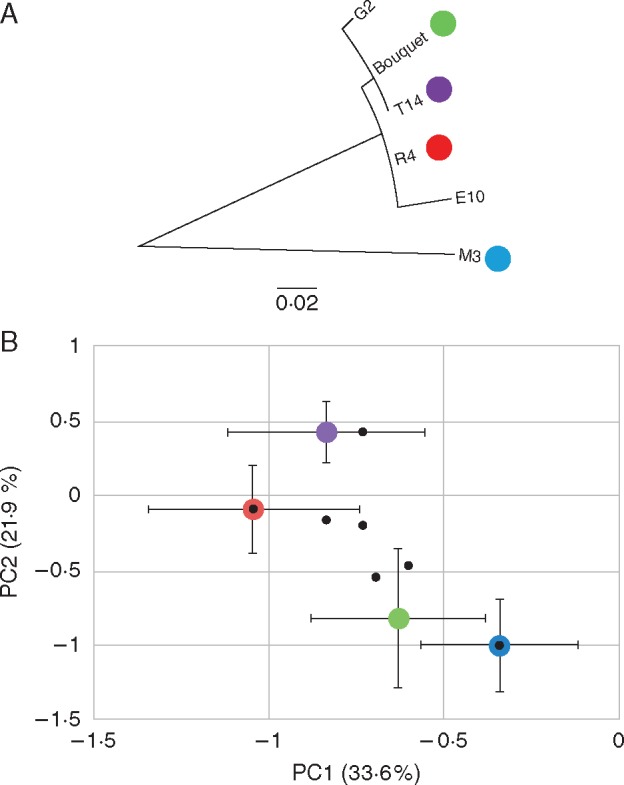
MBSP isolate M3 demonstrates genome-wide diversification based on isolate-specific polymorphisms that are associated with disease symptoms. (A) Maximum likelihood tree of all genomic polymorphic sites among Brazilian MBSP isolates suggests sympatric genetic drift of Piracicaba isolates M3, T14, E10 and R4 rather that diversification by geographic distance compared with G2 and Bouquet, obtained in Guaíra. (B) Allelic polymorphisms in coding regions are associated with specific MBSP isolates with putative effects on disease symptom development. PC1 and PC2 describe >50 % of the symptomatic variation correlated with MBSP genotypes tested. Error bars are one standard error of the mean PC1 and PC2 contribution by each MBSP isolate averaged across all maize genotypes tested. Black dots represent relative PC contribution of polymorphisms in coding sequences from the MBSP isolates.

To investigate what genetic differences between the isolates may contribute to the MBSP-induced branching symptoms, we resequenced the entire genomes of the three other Brazilian MBSP isolates, T14, R4 and Bouquet, that were included in the analyses of the symptoms, and two additional isolates, E10 and G2. Read coverage for all the isolate genomes was >28× the M3 genome size, indicating a high likelihood of identification of all polymorphisms between M3 and the other other MBSP isolates. The genomes of the MBSP isolates revealed a total of only 86 polymorphisms scattered evenly across the genome and not clustering to PMUs (Fig. 4). The majority of these were synonymous single nucleotide polymorphisms (SNPs) rather than insertions or deletions (Supplementary Data Table S5). Forty-nine per cent of the polymorphic sites were in non-coding intergenic regions, 45 % in coding regions, 5 % in pseudogenes and 1 % in tRNA genes. MBSP isolate M3 has the most polymorphic sites and is the most divergent from all sequenced isolates (Fig. 6A). Isolate M3 and isolates T14, E10 and R4 are from from maize fields in Piracicaba, whereas isolates Bouquet and G2 are from maize fields in Guaíra (Table 1). Thus, geographic distance between isolates does not correlate with the number of polymorphisms.

**Fig. 7.**
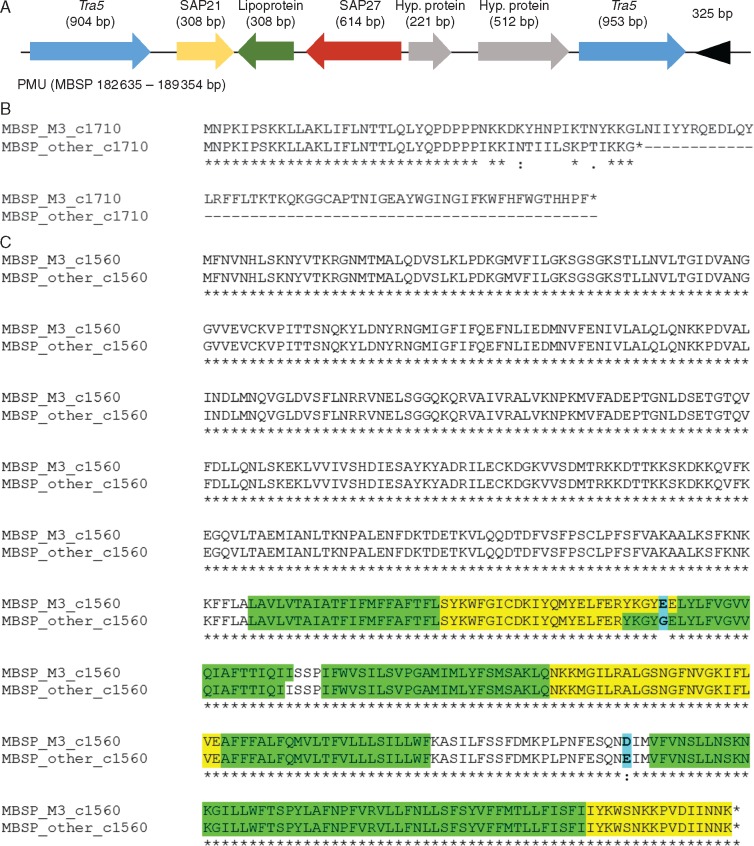
MBSP putative mobile unit (PMU) contains homologues to AY-WB effectors SAP21 and SAP27 (locus tags c1700 and c1720, respectively) as well as a candidate lipoptrotein effector (locus tag c1710) and two other hypothetical proteins, flanked by *tra5* and an inverted 325 bp repeat sequence (A). Alignments of (B) the putative lipoprotein (locus tag c1710) shown in A, and (C) the ABC-type transporter (locus tag c1560) from MBSP isolates and other phytoplasmas. Frameshift in the M3 allele of the putative lipoprotein results in elongation of the encoded peptide sequence compared with other MBSP isolates. The ABC-type transporter has point mutations at the C-terminal part of the protein resulting in predicted alteration of the transmembrane domain folding (TMHMM2·0; green, transmembrane domains; yellow, intracellular domains; white, extracellular domains; blue bold, point mutations).

Only one SNP was found to affect a candidate effector gene and resulted in a frameshift that produces a protein of 103 amino acids in MBSP isolate M3 compared with proteins of 47 amino acids in the other five isolates (locus tag c1710, Table S5). This effector gene lies within MBSP-PMU3 (Fig. 7A, B; Table S4) and is annotated as encoding a phase-variable surface lipoprotein. MBSP-PMU3 also contains sequences similar to candidate effector protein genes SAP21 and SAP27 of AY-WB phytoplasma (Fig. 7A). The lipoprotein, SAP21 and SAP27 genes are about 100 kb apart in the AY-WB genome, but lie adjacent to pseudogenes that are found in PMU-like regions or that are present at high copy numbers in phytoplasma genomes (Bai *et al.*, 2006). Homologues of the SAP21 and SAP27 genes are also found in the genomes of Peanut witches’ broom phytoplasma (PnWB) and *Echinacea purpurea* witches’ broom phytoplasmas, which belong to the 16Sr-II phytoplasma group and are likely to be exchanged PMU elements via horizontal gene transfer with 16Sr-I phytoplasmas (Chung *et al.*, 2013; Ku *et al.*, 2013). Thus, MBSP genomes carry PMU-like pathogenicity islands, and a candidate lipoprotein effector gene on MBSP-PMU3 is polymorphic among Brazilian MBSP isolates.

### Differences in disease symptoms are associated with polymorphic sites in MBSP genomes

We investigated which of the 86 polymorphisms are associated with the MBSP isolate-dependent symptom differences of infected maize genotypes. We first analysed the polymorphisms that correspond to non-synonymous mutations. We identified nine non-synonymous polymorphisms that are unique to MBSP isolate M3 among the MBSP isolates (Table S5) and that may therefore be responsible for segregation of isolate M3 in the symptom PCA (Fig. 6B). One polymorphism involves a G at position 184 853 of the M3 genome that is missing in the other isolates, causing a frameshift change of the candidate lipoprotein effector (locus tag c1710, as discussed above) that produces a longer protein in MBSP isolate M3 compared with the other isolates (102 vs. 47 amino acids, Fig. 7B). This lipoprotein is predicted to be exported by the phytoplasma. Two additional non-synonymous SNPs locate at positions 163 397 and 163 746 within a gene encoding a 536 amino acid conserved ABC-family transporter (locus tag c1560), which has a lipoprotein transporter ATP-binding subunit LolD domain (Blastp E-value: 1·38e-82 against the non-redundant GenBank database). LolD mediates lipoprotein detachment from cytoplasmic membranes (Yakushi *et al.*, 2000). One of the two SNPs in the LolD transporter results in an E/G amino acid change predicted to affect the topology of transmembrane and intracellular regions of the ABC transporter (Fig. 7C). Other non-synonymous polymorphisms in the M3 isolate vs. all the other five isolates are located in genes for enzymes DnaN (F/L amino acid change at base pair 3121), HsdR (N/H amino acid change at base pair 142 611), UvrA (S/A amino acid change at base pair 264 563), Eno (F/L amino acid change at base pair 323 264), Udk (Y/S amino acid change at base pair 483 470) and an ABC transporter fragment that is a predicted pseudogene in all six isolates (frameshift change at base pair 291 106). In addition, nine non-synonymous polymorphisms in isolate M3 were shared with one or more of the other isolates. These were in genes for six enzymes, two ABC transporters and one hypothetical protein (Table S5). Because extracellular lipoproteins and lipoprotein ABC transporters are shown to be virulence factors in other bacteria (e.g. Casabona *et al.*, 2013), we hypothesize that changes in the candidate lipoprotein effector and LolD-like lipoprotein ABC exporter are likely to have the largest impact on the symptom induction differences of M3 vs. the other isolates. However, we cannot rule out the possibility that the amino acid changes in the enzymes and pseudogene differentially impact other aspects such as overall phytoplasma growth in maize plants.

The MBSP isolate Bouquet also induced lateral branching, though this was more dependent on the maize genotype compared with isolate M3, and symptom PCA showed that isolates M3 and Bouquet group more closely together than they did with the other two MBSP isolates (Fig. 6B). However, there were no non-synonymous polymorphisms shared between isolates M3 and Bouquet (Table S5), indicating that polymorphisms in non-coding regions may also impact MBSP symptom induction. To investigate this, we performed genome-wide association tests of all polymorphic loci across the MBSP genomes with variation in disease symptoms across biological replicates of infected maize. We grouped all polymorphic loci according to the combination of allelic variants among the four isolates (Table S5) and then calculated the significance of association between the sets of similar allelic combinations with the quantitative disease traits (lateral branching, height, etc.) in different maize lines and hybrids. Interestingly, in many maize genotypes, lateral branching and cob number are significantly associated with a particular allelic combination (Table 3; polymorphisms 4 and 5) that points to a single locus at base pair 193 568 in the M3 genome that is 83 bp upstream of the start codon of a coding sequence annotated as a 227 amino acid hypothetical protein (M3 genome locus tag c2680) and 47 bp downstream of the stop codon of *lplA* (lipoate-protein ligase A; M3 genome locus tag c2670). Both M3 and Bouquet isolates have a 2 bp insertion at this locus that is absent in the T14 and R4 genomes. The 2 bp insertion may impact the expression levels and transcript stability of the hypothetical protein gene and *lplA*. The hypothetical protein and lipoate-protein ligase A are conserved among phytoplasmas. The hypothetical protein has no known domains, but is a candidate membrane-associated protein based on the presence of six predicted transmembrane domains. Other significant associations we found for other combinations of alleles (Table 3; polymorphism types 1, 2 and 3) point to whole sets of polymorphisms shared between MBSP isolates M3 and R4 (Table S5). There were no obvious symptom differences among MBSP isolate R4, Bouquet and T14, and therefore polymorphisms shared solely between MBSP isolates M3 and R4 are unlikely to have major contributions to symptom development.

**Table 3:** Regression analysis reveals significant association between polymorphic sites in the MBSP genome and disease symptom development

Maize genotype	MBSP polymorphism	Association with MBSP disease symptoms and PCA scores (*P*-value)					
		Lateral branching	No. of internodes	Reddening	No. of cobs	PC1	PC2
2B433PW	1	0·09756	0·8534	0·07515	**0·03407**	0·0634	**0·04504**
	2	0·3621	0·1501	0·1581	0·7609	0·2077	0·8766
	3	0·4978	0·3131	0·7041	0·1147	0·1	0·1047
	4	0·8804	0·5025	**0·02823**	1	0·6864	0·6251
	5	0·108	0·7388	1	0·05977	0·1704	0·1704
30F35H	1	0·06608	0·4714	0·445	0·4421	0·6377	0·2414
	2	0·6556	0·1578	0·194	0·7997	0·6736	0·8046
	3	0·2548	0·5721	0·6616	0·6595	0·4363	0·4329
	4	0·2461	0·2781	0·869	0·2332	0·9886	0·2054
	5	**0·008789**	0·9307	0·5711	0·1239	0·6667	**0·04529**
CRE1	1	**0·0118**	0·3166	0·9026	0·1627	0·03106	0·1342
	2	0·5119	0·08189	0·0842	0·4401	0·5228	0·9889
	3	0·1765	**0·01073**	0·1561	0·6587	0·2629	0·2234
	4	1	0·5338	0·08605	0·693	0·1	0·6251
	5	**0·03726**	0·7125	0·1616	0·1313	0·6125	0·3839
CRE2	1	0·4647	0·3863	0·362	0·5786	0·3461	0·9021
	2	0·8302	0·6761	0·714	0·06042	**0·01636**	0·8326
	3	0·6512	0·252	0·2549	0·2698	0·2512	0·938
	4	**0·01727**	0·4637	0·006386	**0·008621**	0·07291	**0·00546**
	5	**0·0132**	0·1814	0·308	**0·01409**	**0·02413**	**0·03838**
CRE3	1	**0·0143**	0·5274	0·5966	0·05989	0·274	**0·01925**
	2	0·1145	1	1	0·556	0·8973	0·2144
	3	0·3924	0·5628	0·6278	0·2317	0·3721	0·3025
	4	0·8655	0·6301	1	0·3741	**0·04475**	0·6814
	5	**0·01723**	0·3306	0·6278	**0·01022**	**0·00534**	**0·01139**

Maize phenotypic traits that were significantly influenced by MBSP infection as well as interactions between maize and pathogen genotypes (Table 2) were analysed for association with polymorphic differences among MBSP isolates using single-marker regression analysis.

We identified five different polymorphic combinations at any given site (Table S5) and regressed the genotype with disease symptoms as quantitative traits.

Certain combinations of MBSP polymorphisms significantly correlate with lateral branching, number of cobs and internodes as well as PCA scores describing correlated changes in symptomatic traits.

In summary, only a few polymorphic sites in the genomes of the MBSP isolates explain the symptom differences induced by the MBSP isolates during infection of maize genotypes. A candidate lipoprotein effector protein and lipoprotein ABC export protein are putative virulence factors that we found to be associated with the lateral branching phenotype during MBSP infection of maize.

## DISCUSSION

Infection by MBSP induces multiple morphological alterations in the maize host plant and we found that MBSP isolates from two maize-growing regions of South-east Brazil differ in the strength of disease symptom induction, including lateral branching. By whole-genome sequencing we identified 86 polymorphic sites among multiple MBSP isolates, of which 45 % are located in protein-coding regions. Nine polymorphisms are associated with the lateral branching symptoms, of which three polymorphisms are located within coding sequences of two candidate virulence factors, which are a phase-variable lipoprotein and an ATP-dependent lipoprotein ABC export protein.

Symptom analyses and genome sequencing of multiple MBSP isolates provides a way to find sites that are under selection and are therefore likely to be involved in MBSP pathogenicity, consistent with other studies that have shown that genetically monomorphic plant pathogens contain sites that are under strong selection for their function in virulence and evasion of host immune recognition (Cai *et al.*, 2011). MBSP isolates group based on whether they induce lateral branching or necrosis symptoms in maize (Fig. 3), and the latter is dependent on maize genotype. Maize hybrids 2B433PW and 30F35H analysed in this study are commonly grown in South-east Brazil and hence it is likely that there is strong selection for MBSP to increase virulence on these maize genotypes. Necrosis could be indicative of immune recognition of MBSP by the plant, whereas the lateral branching symptoms are advantageous for the phytoplasma. Consistent with this is that phytoplasmas and their insect vectors are biotrophs requiring live tissue for replication, and necrosis may therefore be a plant defence response to restrict pathogen growth and insect vector colonization. In contrast, the lateral branching symptoms may promote MBSP phytoplasma fitness. For example, bushier plants promote insect vector colonization directly or indirectly (Sugio *et al.*, 2011*a*) and could provide more sink tissue (such as young leaves) where phytoplasma can replicate. Clonal variants of MBSP that aid vector performance could be spread quickly over long distances by the migratory *D. maidis*. There are differences in transmission efficiencies of MBSP and AY phytoplasma isolates (Murral *et al.*, 1996; Moya-Raygoza and Nault, 1998), and a dynamic spatio-temporal structure of MBSP isolates in the field is suggested by our observation that divergent MBSP isolates were collected from the same maize field in the same year. Moreover, *D. maidis* is capable of long-distance migration and can also survive locally, probably on alternative plant hosts, when maize plants are absent (Oliveira *et al.*, 2007, 2013; E. Oliveira *et al.*, 2007; Moya-Raygoza *et al.*, 2007). Further studies are required to assess if the necrosis vs. lateral branching symptoms impact MBSP fitness either directly by increasing phytoplasma titres in maize plants or indirectly via promoting leafhopper attraction, migration and transmission.

Bacterial lipoproteins can have diverse virulence functions. They may be perceived as pathogen-associated molecular patterns (PAMPs) by host pattern recognition receptors (PRRs) (Janeway and Medzhitov, 2002) such as extra- or intracellular TIR (Toll/interleukin-1) domains or NOD family receptors to trigger immune responses in both plants and animals (Medzhitov, 2001). For example, mycoplasmal lipopeptide MALP2 is recognized by a TLR2 receptor (Takeuchi *et al.*, 2000). Lipoproteins are also implicated in a wide range of invertebrate immune responses, including activation of antifungal and antibacterial responses (Whitten *et al.*, 2004). These proteins have a role in cellular adhesion (Paredes *et al.*, 2015) and the recruitment (transport) of host lipids (Herren *et al.*, 2014). While in Gram-negative bacteria several ABC transporter subunits (LolCDE) are required for lipoprotein detachment from the inner membrane (Yakushi *et al.*, 2000), phytoplasmas have only a single lipid membrane and no cell wall and therefore may require only one ABC transporter subunit, such as LolD, to export a lipoprotein. Interestingly, in *Pseudomonas aeruginosa*, a LolD-type ABC exporter and an exported lipoprotein promote the activity of the type VI secretion system (Casabona *et al.*, 2013). Even though phytoplasmas lack components that are characteristic of Type III, IV and VI secretion systems, an intriguing possibility arises that the lipoprotein effector and ABC exporter are involved in the attachment of phytoplasma cells to host cells and the activation of the secretion of other candidate effector proteins, including perhaps MBSP SAP11 and tengu-su homologues. SAP11 of AY-WB phytoplasma and tengu-su of OY-M induce lateral branching symptoms in plants (Hoshi *et al.*, 2009; Sugio *et al.*, 2011*a*). AY-WB SAP11 interacts with and destabilizes *A. thaliana* TCP family members that regulate lateral branching (Sugio *et al.*, 2011*a*, 2014). Hence, MBSP SAP11 may destabilize the maize TCP transcription factor TEOSINTE BRANCHED1 (TB1), which controls apical dominance and suppression of both basal (tillering) and lateral branching (Doebley *et al.*, 1997; Whipple *et al.*, 2011). However, the functions of MBSP effectors have not yet been characterized. Moreover, it is possible that the lipoprotein has a direct effect on plant symptom development. For example, lipoproteins on the surface of phytoplasma may be recognizd by plant receptors that also function in organ patterning and development (De Smet *et al.*, 2009), possibly inducing morphological changes in plant architecture.

## Conclusions

There is probably strong selection on MBSP to increase virulence and insect transmission on maize genotypes. MBSP isolates collected from two maize-growing areas in Brazil show differences in lateral branching and necrosis symptoms. These symptom differences are associated with polymorphisms in a phase-variable lipoprotein, which is a candidate effector, and an ATP-dependent lipoprotein ABC export protein, whereas no polymorphisms were observed in other candidate effector genes. Lipoproteins and ABC export proteins of other pathogens activate host defence responses, regulate pathogen attachment to host cells and activate effector secretion systems. Hence, it is possible that the MBSP virulence proteins promote lateral branching directly or indirectly via activation of the SAP11 and tengu-su effector, which both induce lateral branching of plants (Hoshi *et al.*, 2009; Sugio *et al.*, 2011*a*).

## SUPPLEMENTARY DATA

Supplementary data are available online at www.aob.oxfordjournals.org and consist of the following. Table S1: maize genotypes and hybrids inoculated with MBSP isolates for phenotypic analysis. Table S2: primer pairs used for detection of MBSP, the effector proteins in the pathogen SAP island and the reference gene of maize. Table S3: list of all predicted effectors from the fully assembled genome sequence of MBSP. Table S4: list of all predicted MBSP effectors and their distance from PMU-like genes. Table S5: list of all polymorphisms among MBSP isolates M3, R4, T14 and Bouquet. Figure S1: MBSP induces leaf reddening, yellowing and necrotic lesions but does not affect internode number. Figure S2: Mexico and Brazil MBSP isolates demonstrate complete conservation of SAP11 effector protein homologue and share similar arrangement of coding sequences within the SAP11 genomic island.

## Supplementary Material

Supplementary Data
