# Supplementary material for: A few sequence polymorphisms among isolates of Maize bushy stunt phytoplasma associate with organ proliferation symptoms of infected maize plants
Source: Ann Bot. 2016 Dec 10;119(5):869–84. doi: 10.1093/aob/mcw213 (PMC5379588; doi:10.1093/aob/mcw213)
Supplement: Supplementary Data [file mcw213_Supp.docx]

**Supplementary Information**

Table S1. Maize genotypes and hybrids inoculated with MBSP isolates for phenotypic analysis

| **Maize Line/Hybrid** | **MBSP Isolates** | | | | **Non-inoculated control plants** |
| --- | --- | --- | --- | --- | --- |
|  | **R4** | **T14** | **M3** | **Bouquet** |  |
| CRE1 | 5 | 6 | 4 | 3 | 6 |
| CRE2 | 5 | 6 | 5 | 3 | 6 |
| CRE3 | 4 | 6 | 6 | 4 | 6 |
| 30F35H (Pioneer^TM^) | 4 | 6 | 4 | 2 | 5 |
| 2B433PW (Dow Agrosciences^TM^) | 4 | 6 | 5 | 3 | 6 |

Table S2. Primer pairs used for detection of Maize Bushy Stunt Phytoplasma, the effector proteins in the pathogen Secreted Aster Protein island and the reference gene of maize


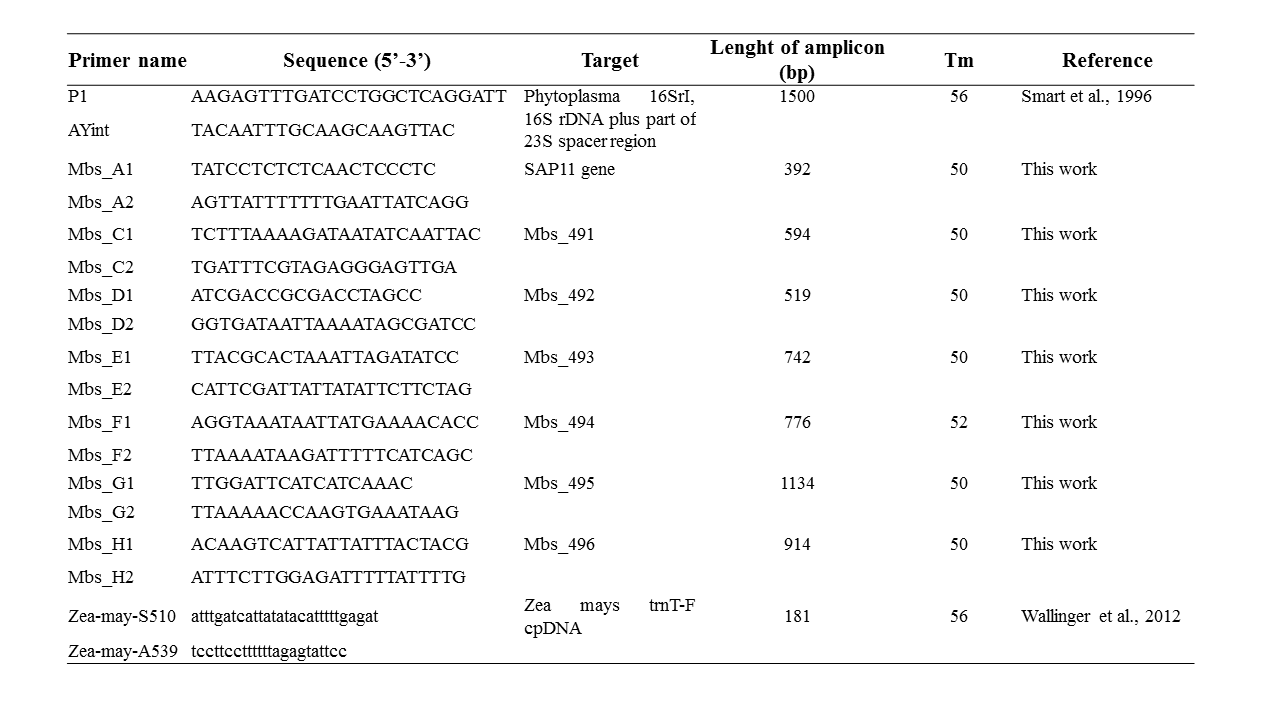


Table S3. List of all predicted effectors from the fully assembled genome sequence of MBSP. M3 isolate shows conservation of effector proteins in MBSP. There are few polymorphic sites in the effector genes or 1000 bp regions up- and down-stream the CDS. Putative effector c1710 has a M3-specific SNP relative to other isolates sequenced.

Table S4. List of all predicted MBSP effectors and their distances (bp) from PMU-like genes (Bai *et al.*, 2006). Positive integers refer to upstream effector position, negative – to downstream location relative to PMU genes. All distances within 20 000 bp (the equivalent size of PMU1 of AY-WB, Toruño *et al*., 2010) are indicated in bold. There are several copies of *tra5* and *sigF* genes in MBSP genomes, and their distances to the closest copy are represented in the table. Effectors that are within 20 kb distance from at least two PMU-like genes are suggested PMU-like clusters and shaded gray. PMU3 cluster contains at least 6 predicted effectors, including SAP21, SAP27, SAP63 homologs and a predicted lipoprotein, see Fig. 5B.


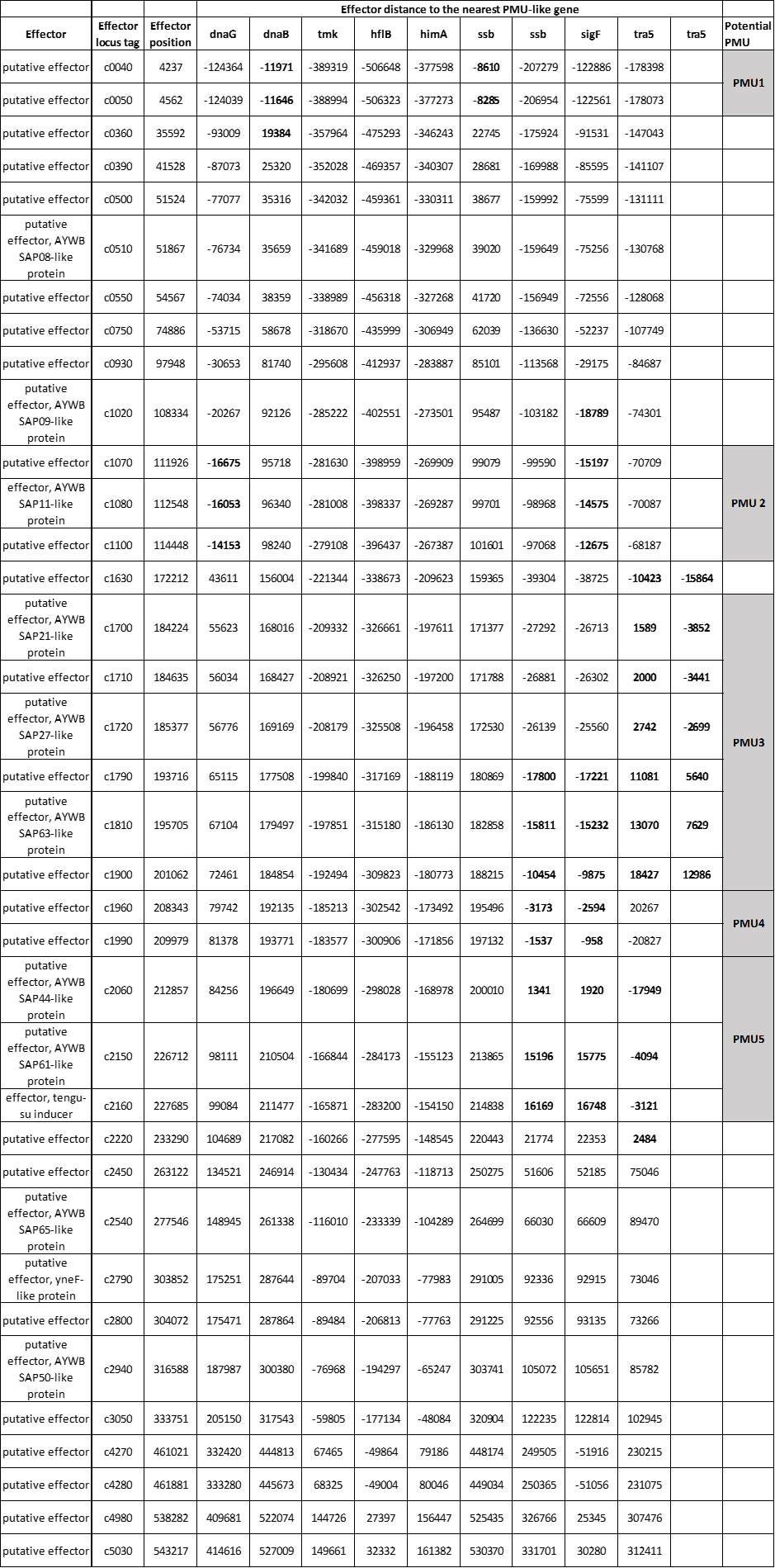


Table S5. List of all polymorphisms among M3, R4, T14 and Bouquet isolates (below). Many polymorphic loci are bi-allelic (the set of type 1 polymorphism), the other loci are tri-allelic. Since the single-marker regression analysis can perform the association test with only bi-allelic loci, all tri-allelic loci were considered as various parsimonious combinations of bi-allelic loci assuming that each locus can be ancestral to the other two as shown below:

B

A

B

C

B

A

B

A

C

A

C

A

|  |  |  |  | **Assumption** | **Convert to biallelic** | | | | **Polymorphism type** |
| --- | --- | --- | --- | --- | --- | --- | --- | --- | --- |
| **Biallelic** | | | |  |  |  |  |  |  |
| **A** | **B** | **B** | **B** | **A=A; B=B** | **A** | **B** | **B** | **B** | **1** |
| **Triallelic** | | | |  |  |  |  |  |  |
| **A** | **B** | **C** | **C** | **A=C; B=B** | **A** | **B** | **A** | **A** | **2** |
|  |  |  |  | **A=B; C=C** | **A** | **A** | **C** | **C** | **3** |
|  |  |  |  | **B=C; A=A** | **A** | **B** | **B** | **B** | **1** |
| **A** | **C** | **B** | **C** | **A=C; B=B** | **A** | **A** | **B** | **A** | **4** |
|  |  |  |  | **A=B; C=C** | **A** | **C** | **A** | **C** | **5** |
|  |  |  |  | **B=C; A=A** | **A** | **B** | **B** | **B** | **1** |

This also allowed us to simulate association probability with the nature of mutation at each given tri-allelic locus. For example in table below, polymorphic locus at the position 293568, we can hypothesise that (1) Bouquet sequence (ancestral) has undergone a step-wise deletion; (2) M3 and Bouquet have insertion compared to ancestral sequence; (3) M3 was the ancestral state which has mutated twice. In either case we assume any mutation in ancestral sequence to be either loss- or gain-of-function (bimodal state).


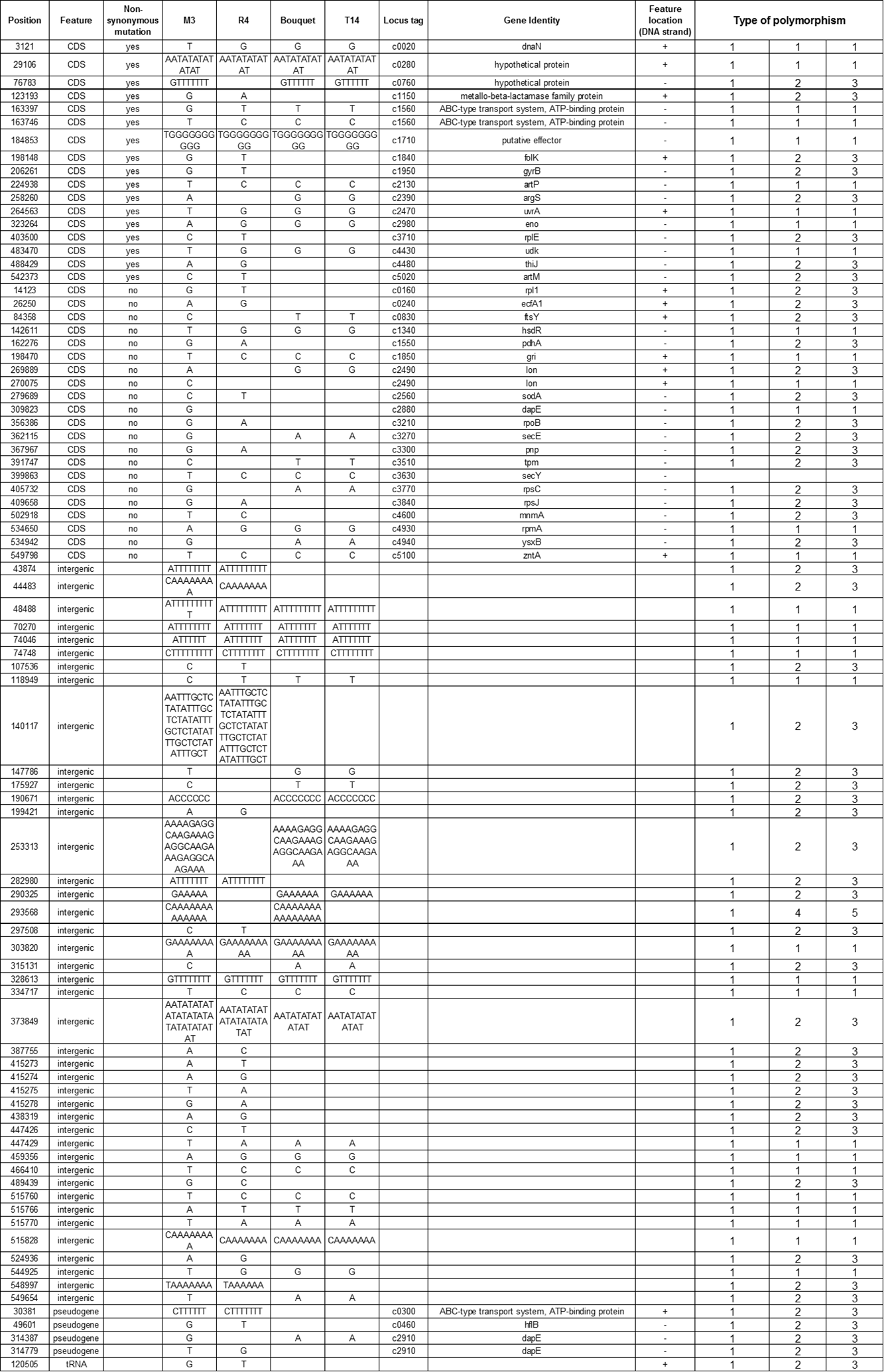


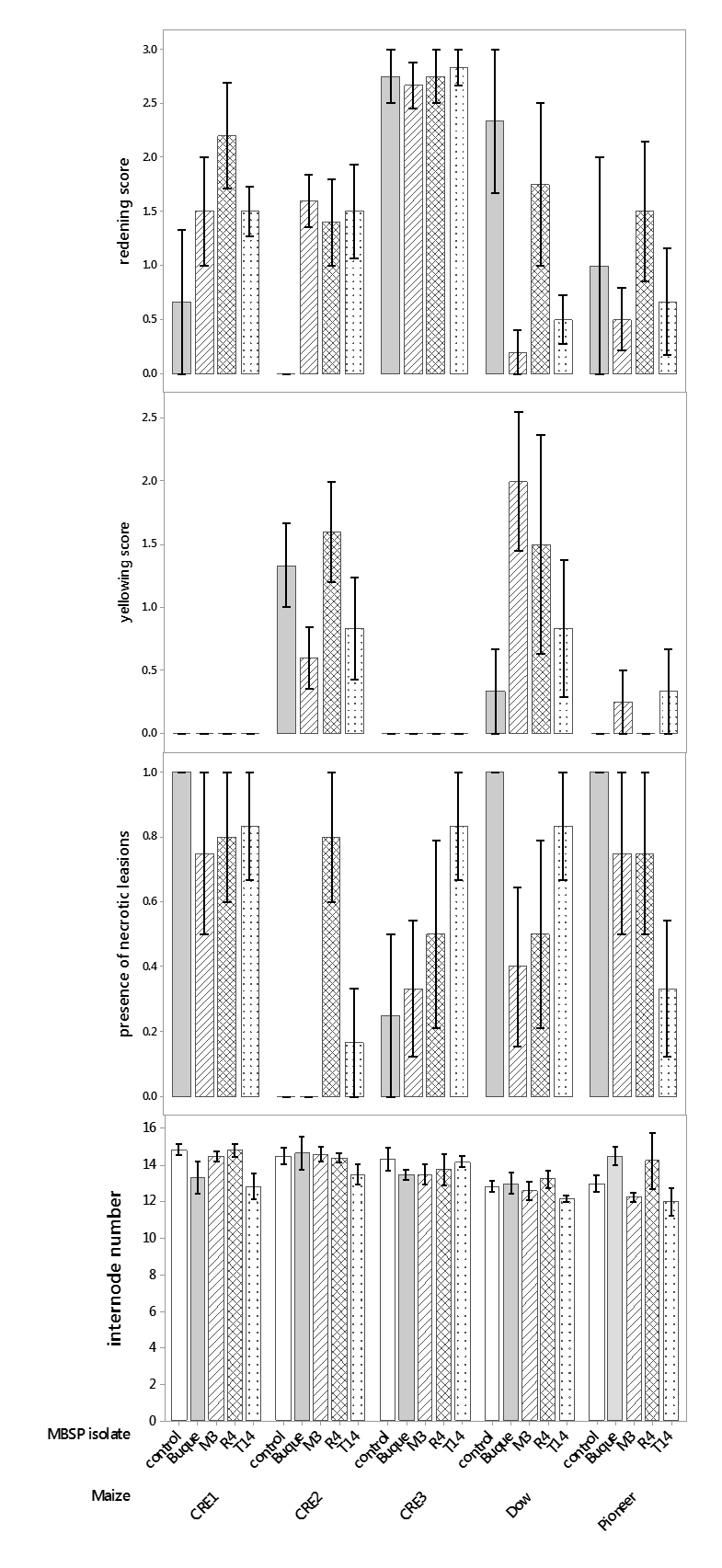


Fig. S1. MBSP induces leaf reddening (A), yellowing (B) and necrotic lesions (C) but does not affect internode number (D). Reddening and yellowing was scored on a relative scale 0 (healthy control) to 3 (severe reddening). Necrosis was scored as present or absent (healthy plant). Healthy plants did not show any change in coloration or necrosis. Internode number number in MBSP-infected maize was compared against the healthy plant.


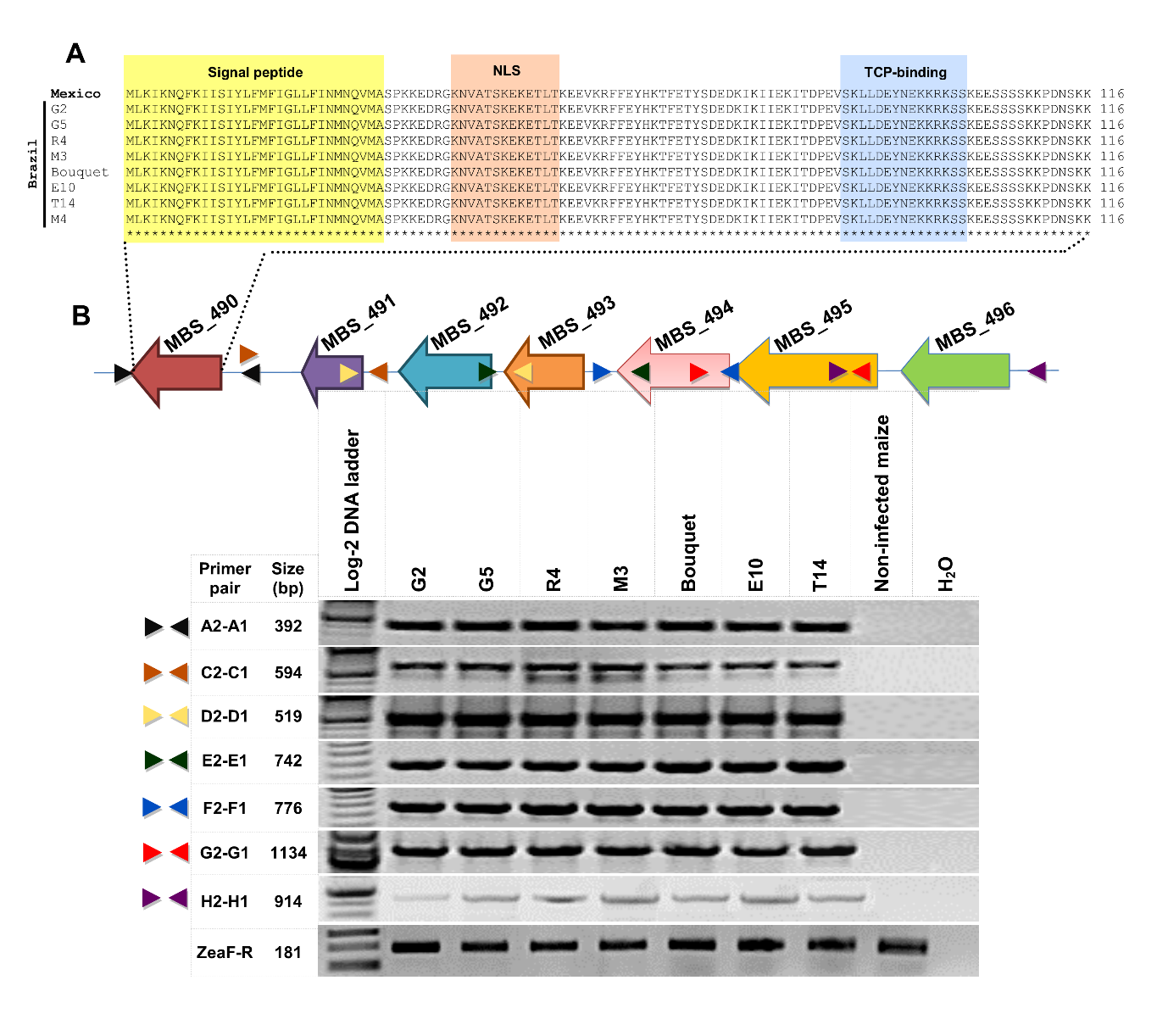


Fig. S2. Mexico and Brazil MBSP isolates demonstrate complete conservation of SAP11 effector protein homolog (A) and share similar arrangement of coding sequences within SAP11 genomic island (B). The signal peptide, nuclear localisation signal (NLS) and TCP-binding domain are highlighted. The SAP11 island (arrow blocks) was recunstructed using the available MBSP genome sequence from the Mexican isolate (Sugio and Hogenhout, 2012) and used for designing primer pairs (Supplementary Information, Table S2) to characterise the potential sequence similarity and the arrangement of coding sequences within SAP11 genomic island.

**Supplementary file references**

**Smart CD, Schneider B, Blomquist CL, Guerra LJ, Harrison NA, Ahrens U, Lorenz KH, Seemüller, Kirkpatrick BC. 1996.** Phytoplasma-specific PCR primers based on sequences of the 16S-23S rRNA spacer region. *Applied and Environmental Microbiology* **62**: 2988-2993.

**Sugio A, Hogenhout SA. 2012.** The genome biology of phytoplasma: modulators of plants and insects. *Current Opinion in Microbiology* **15**: 247-254.

**Wallingeer C, Juen A, Staudacher K, Schallhart N, Mitterrutzner E, Steiner E-M, Thalinger B, Traugott M. 2012.** Rapid plant identification using species- and group-specific primers targeting chloroplast DNA. *PLoS One* **7**:e29473.
